# Fast and Robust Reconstruction for Fluorescence Molecular Tomography via *L*_1-2_ Regularization

**DOI:** 10.1155/2016/5065217

**Published:** 2016-12-06

**Authors:** Haibo Zhang, Guohua Geng, Xiaodong Wang, Xuan Qu, Yuqing Hou, Xiaowei He

**Affiliations:** School of Information Sciences and Technology, Northwest University, Xi'an, Shaanxi 710027, China

## Abstract

Sparse reconstruction inspired by compressed sensing has attracted considerable attention in fluorescence molecular tomography (FMT). However, the columns of system matrix used for FMT reconstruction tend to be highly coherent, which means *L*_1_ minimization may not produce the sparsest solution. In this paper, we propose a novel reconstruction method by minimization of the difference of *L*_1_ and *L*_2_ norms. To solve the nonconvex *L*_1-2_ minimization problem, an iterative method based on the difference of convex algorithm (DCA) is presented. In each DCA iteration, the update of solution involves an *L*_1_ minimization subproblem, which is solved by the alternating direction method of multipliers with an adaptive penalty. We investigated the performance of the proposed method with both simulated data and* in vivo* experimental data. The results demonstrate that the DCA for *L*_1-2_ minimization outperforms the representative algorithms for *L*_1_, *L*_2_, *L*_1/2_, and *L*_0_ when the system matrix is highly coherent.

## 1. Introduction

Fluorescence molecular tomography (FMT) has become a promising molecular imaging modality since it has the ability to provide localization and quantitative analysis of the fluorescent probe for preclinical research [1, 2]. However, FMT reconstruction suffers from high ill-posedness due to the insufficiency of external measurements, which is caused by high absorption and scattering in photon propagation through biological tissues [3].

To alleviate the ill-posedness of FMT, some* a priori* information, such as anatomical information, optical properties, permissible region, and sparsity of target distribution, has been successfully incorporated in FMT reconstruction [4–7]. In addition, many regularization techniques have also been devoted to get an accurate and stable solution. Conventionally, *L*_2_ norm regularizer is a common penalty term in spite of its over-smoothness and results with lower resolution [8]. Another common regularizer is *L*_0_ norm, which is nondeterministic polynomial (NP) hard and can be solved by a greedy approach such as the orthogonal matching pursuit (OMP) [9]. Inspired by compressive sensing (CS) theory, the *L*_1_ norm regularizer as the convex relaxation of *L*_0_ has become a widely used sparsity-inducing norm for FMT reconstruction [10–13]. However, *L*_1_ norm regularizer is not always providing the sparsest solution for the inverse problem of FMT [14]. This gives way to nonconvex *L*_*p*_ (0 < *p* < 1) norm regularizer, which has been applied to optical tomography and was found to have better results than *L*_1_ does [15]. Some comparative studies show that nonconvex *L*_*p*_ (0 < *p* < 1) norm regularizer with *p* near 1/2 performs the best result among regularizers of *L*_*p*_ (0 < *p* < 1) norm [16].

Recently, a new nonconvex regularizer named *L*_1-2_ has been proposed and produced better solution than *L*_*p*_ (*p* = 1/2) norm regularizer when the sensing matrix was large and highly coherent [17, 18]. The Magnetic Resonance Imaging (MRI) image recovery tests have also indicated that *L*_1-2_ norm regularizer outperforms *L*_1/2_ and *L*_1_ for highly coherent matrix [17]. Meanwhile, the columns of system matrix used for FMT reconstruction are also highly coherent with the finite element computing framework [19].

In this paper, new *L*_1-2_ norm regularization was proposed to improve the FMT imaging. In our method, a difference of convex algorithm (DCA) was presented to solve the nonconvex *L*_1-2_ minimization problem. And the alternating direction method of multipliers (ADMM) with an adaptive penalty was used to solve the subproblem with fast convergence for each DCA iteration. The performance of the proposed method was validated with simulated data and* in vivo* experimental data.

The outline of this paper is as follows.  Section 2 elaborates the forward model and *L*_1-2_ norm regularization algorithm.  Section 3 demonstrates the feasibility and effectiveness of the method with both simulated data and* in vivo* experimental data. Finally, we conclude the paper and discuss relevant issues in Section 4.

## 2. Methods

### 2.1. Light Propagation Model

As an approximation to Radiative Transfer Equation (RTE), the Diffusion Approximation associated with Robin boundary conditions has been widely used for modeling the light transportation in biological tissues [20, 21]. For steady-state FMT with point excitation sources, the coupled diffusion equations can be presented as follows:

(1) ∇·Dexr∇Φexr−μa,exrΦex=−Θδr−rs∇·Demr∇Φemr−μa,emrΦem=−Φexrημafr,

where subscript ex and em denote excitation light and emission light, respectively. *r* ∈ *Ω* is the domain under consideration. *D*_ex_ = 1/3(*μ*_*a*,ex_ + *μ*_*s*,ex_′) and *D*_em_ = 1/3(*μ*_*a*,em_ + *μ*_*s*,em_′) are diffusion coefficients with *μ*_*a*,ex_, *μ*_*a*,em_ as absorption coefficients for excitation and emission wavelengths, *g* is the anisotropy parameter, and *μ*_*s*,ex_′, *μ*_*s*,em_′ are the reduced scattering coefficients. Φ_ex_ and Φ_em_ denote the photon density. *ημ*_*af*_ is the unknown fluorescent yield to be reconstructed. Using the finite elements method (FEM), (1) can be linearly discretized as follows:

(2) KexΦex=bexKemΦem=FX,

where *K*_ex_ and *K*_em_ denote the system matrix at excitation and emission wavelengths, respectively. The symmetric matrix *F* is obtained by discretizing the unknown fluorescent yield distribution. The final linear relationship between the unknown fluorescence yield *x* and the measured surface data *ϕ* can be obtained as follows:

(3) $$\begin{array}{c} Ax=\phi , \end{array}$$

where *A* is *M* × *N* linear system matrix which is large-sized and ill-posed.

### 2.2. Inverse Reconstruction of FMT by DCA-*L*_1-2_ Algorithm

The CS theory provides sufficient conditions for the exact recovery of the sparse signals from limited number of measurements. One commonly used concept is the mutual coherence [17, 22] which is defined as

(4) $$\begin{array}{c} \mu \left(\;A\;\right)=\underset{i\neq j}{\max}\frac{\left|a_{p}^{T}\cdot a_{q}\right|}{{\left\|a_{p}\right\|}_{2}\cdot {\left\|a_{q}\right\|}_{2}}, \end{array}$$

where *a*_*p*_ and *a*_*q*_ are different columns of *A*. The mutual coherence of system matrix *A* derived by FEM method is always as high as above 90% [19]. In the highly coherent regime of CS, *L*_*p*_ (*p* = 1/2) and *L*_1-2_ norm regularizers are expected to yield the sparest solution that *L*_1_ regularization always fails to [17, 18].

Recently, a DCA-*L*_1-2_ algorithm was proposed and theoretical properties of *L*_1-2_ minimization have been proved in papers [17, 23]. Considering the advantages of *L*_1-2_ minimization, we converted linear matrix equation (3) into the following unconstrained optimization problem:

(5) $$\begin{array}{c} \underset{x\in R^{n}}{\min}\;\frac{1}{2}{\left\|\;Ax-\phi \;\right\|}_{2}^{2}+\lambda \left(\;{\left\|\;x\;\right\|}_{1}-{\left\|\;x\;\right\|}_{2}\;\right), \end{array}$$

where *λ* > 0 is a regularization parameter which is usually empirically selected and ‖*x*‖_1_ − ‖*x*‖_2_ denotes the *L*_1-2_ regularization operator.

To resolve minimization problem (5), the difference of convex algorithm (DCA) [24] which is a descent method without line search was used. Equation (5) can be decomposed into DC decomposition as *F*(*x*) = *G*(*x*) − *H*(*x*), where

(6) Gx=12Ax−ϕ22+λx1,Hx=λx2.

In (6), ‖*x*‖_2_ is differentiable with gradient *x*/‖*x*‖_2_. An iterative scheme was used to solve *F*(*x*) as follows:

(7) xn+1=arg minx∈Rn⁡ 12Ax−ϕ22+λx1−x−xn,xnxn2.

In each DCA iteration, there is a *L*_1_-regularized convex subproblem that needs to be solved:

(8) $$\begin{array}{c} \underset{x\in R^{n}}{\min}\;\frac{1}{2}x^{T}\left(\;A^{T}A\;\right)x+{\left(\;x^{T}\phi +\lambda \frac{x^{n}}{{\left\|x^{n}\right\|}_{2}}\;\right)}^{T}x+\lambda {\left\|\;x\;\right\|}_{1}. \end{array}$$

We use the augmented Lagrangian method and transform (8) into the following:

(9) Lδx,y,u=12xTATAx+ATϕ+λxnxn2Tx+λy1+uTx−y+δ2x−y22.

The subproblem is solved by minimizing *L*_*δ*_ with respect to *x*, minimizing *L*_*δ*_ with respect to *y*, and updating *u* successively. In order to solve (9) with a fast speed of convergence, an ADMM strategy with an adaptive penalty [25] was utilized as follows:

(10) xk+1=arg minx⁡ Lδkx,yl,ul,yk+1=arg minz⁡ Lδkxl+1,y,ul,uk+1=ul+δkxl+1−yl+1,δk+1=min⁡δmax,ρδk.

In the above iterations, the update of *y* is based on the soft-thresholding operator, where

(11) $$\begin{array}{c} {\left(\;S\left(\;x,r\;\right)\;\right)}_{i}=sgn\left(\;x_{i}\;\right)\max\left\{\;\left|\;x_{i}\;\right|-r,0\;\right\}. \end{array}$$

Meanwhile, the penalty *δ* was updated as an adaptive form as follows:

(12) $$\begin{array}{c} \delta_{k+1}=\min\left(\;\delta_{max},\rho \delta_{k}\;\right), \end{array}$$

where *δ*_max_ is an upper bound of {*δ*_*k*_} and *ρ* is defined as follows:

(13) $$\begin{array}{c} \rho =\left\{\begin{array}{cc} \rho_{0}, & \text{if }\frac{\delta \left\|y^{k+1}-y^{k}\right\|}{x^{k+1}}<\varepsilon \\ 1, & \text{otherwise,} \end{array}\right. \end{array}$$

where *ρ*_0_ ≥ 1 is a constant.

Algorithm 1 presents the iterative process of DCA-*L*_1-2_ algorithm for FMT reconstruction. To begin with the iteration, the initial value *x* ^1^ was set as *L*_1_ subproblem.

## 3. Experiments and Results

In this section, the simulations both on 3D digital mouse model and* in vivo* experiments were used to demonstrate the potential and feasibility of the DCA-*L*_1-2_ algorithm for FMT reconstruction. To investigate the performance of DCA-*L*_1-2_ algorithm, four representative regularizers, including *L*_1/2_, *L*_1_, *L*_2_, and *L*_0_, were used for a systematical comparison. More specifically, iterative reweighted least squares algorithm (IRLS-*L*_1/2_) [26], incomplete variables truncated conjugate gradient algorithm (IVTCG-*L*_1_) [27], Tikhonov regularization algorithm (Tikhonov-*L*_2_) [28], and OMP algorithm [29] were compared with the DCA-*L*_1-2_ algorithm, respectively, as the corresponding method for the above regularizers.

The qualities of reconstruction results are quantitatively evaluated in terms of the absolute location error (LE) [3], reconstructed fluorescent yield (Recon. FY) [3], normalized root mean square error (NRMSE) [16], the percentage of nonzero coefficient (PNZ) [16], and time cost. The experiment codes were written in MATLAB and were performed on a desktop computer with 3.40 GHz Intel® Xeon® Processor E3-1231 and 12 G RAM.

### 3.1. Numerical Simulation Experiments

**Figure 1:**
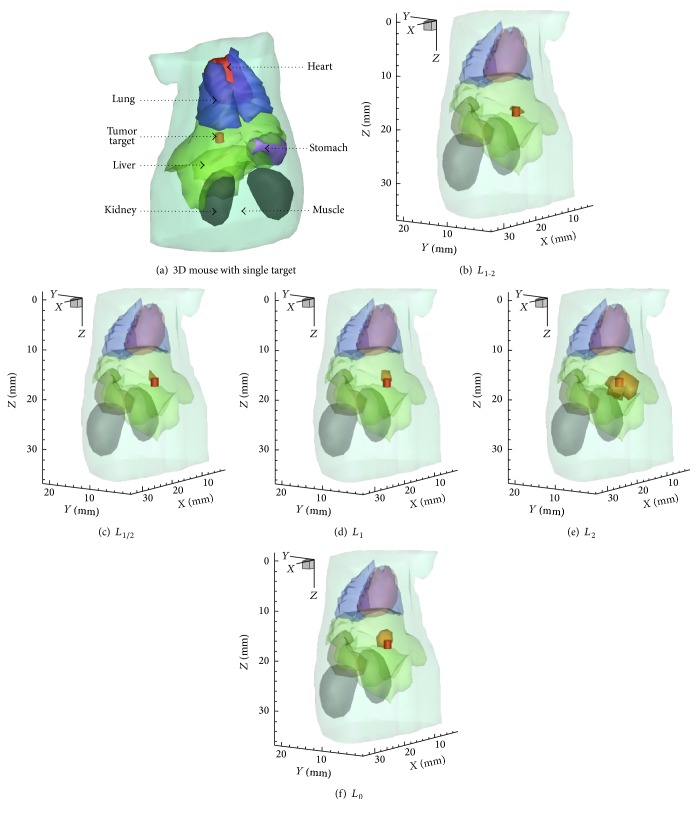
The mouse model and the 3D views of the reconstructed results. (a) The mouse model with single target. (b–f) The 3D views of the reconstructed results where the red cylinder is the real fluorescent target.

**Figure 2:**
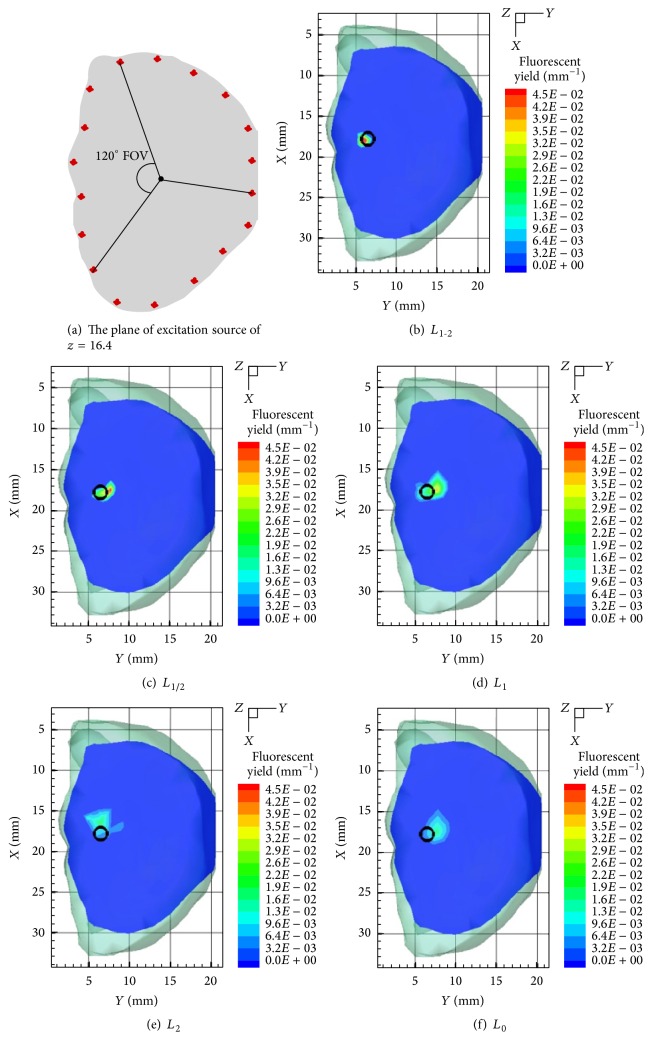
The plane of 18 excitation sources and the 2D views (*Z* = 16.4 mm) of the reconstructed results. (a) The plane of 18 excitation sources with 120° FOV. (b–f) The 2D views (*Z* = 16.4 mm) of the reconstructed results by six comparative methods. The black circle denotes real position of fluorescent target.

A 33 mm height torso extracted from a 3D mouse atlas was utilized to simulate the heterogeneity of biological tissues [30].  Figure 1(a) shows the mouse model with six organs.  Table 1 lists the specific optical parameters. A cylinder with a radius of 0.8 mm and a height of 1.6 mm was positioned at 17.8, 6.6, and 16.4 mm to mimic the fluorescent target. The actual fluorescent yield was set to be 0.05 mm^−1^. For excitation, we used 18 excitation sources being located on the plane of *Z* = 16.4 mm as shown in Figure 2(a). The surface data on the opposite side with a 120° field of view (FOV) were measured for each excitation source. A total of 18 datasets were assembled for the subsequent reconstruction process.

The forward FEM mesh was discretized into 24231 nodes and 128300 tetrahedral elements. Meanwhile, the FEM mesh for inverse reconstruction was discretized into 2601 nodes and 12752 tetrahedral elements. The mutual coherence of the system matrix for inverse reconstruction was 99.96%.

Figures 1(b)–1(f) present a comparison of reconstruction results with 3D views for single fluorescent target. The corresponding 2D section-views on the excitation plane are demonstrated in Figures 2(b)–2(f).  Table 2 gives the quantitative results of the five regularization methods.

As shown in Figures 1(b)–1(f), Figures 2(b)–2(f), and Table 2, reconstruction results of *L*_1-2_ and *L*_1/2_ were remarkable. Compared to the other three methods, results of DCA-*L*_1-2_ and IRLS-*L*_1/2_ have fewer artifacts, lower LE, lower NRMSE, and lower PNZ. Meanwhile, the Recon. FY by DCA-*L*_1-2_ and IRLS-*L*_1/2_ were closer to 0.05 mm^−1^. The proposed DCA-*L*_1-2_ completely outperforms the other methods, except for a slightly larger time consumption compared to OMP.

Generally speaking, the quality of FMT reconstruction is influenced by the number of excitation sources. To test the stability of the algorithm, different numbers of excitation sources were used for reconstruction.  Table 3 gives the corresponding reconstructed results with 18, 12, 8, and 4. Obviously, the decreased number of excitation sources leads to significant reduction of measurements. Nevertheless, the results of DCA-*L*_1-2_ are generally satisfactory even in the case of 4 excitation sources.

The quality of reconstructed results is sensitive to measurement noise because of the severe ill-condition of system matrix. For stability test, four different levels of Gaussian noise (5%, 15%, 25%, and 35%) were added to the synthetic measurements.  Table 4 shows the reconstruction results under 4 different noise levels. It shows that the DCA-*L*_1-2_ algorithm is quite resilient with Gaussian noise.

### 3.2. *In Vivo* Evaluation with Implanted Fluorophore

The performance of DCA-*L*_1-2_ and IRLS-*L*_1/2_ was remarkably compared to the other three methods in the simulation experiments. In this section, we further evaluated the performance of the proposed algorithm with* in vivo* experimental data [8].

In this experiment, an adult BALB/C mouse with a glass tube implanted into its abdomen was used. The experimental data was acquired by a hybrid FMT/Micro-CT system [8]. The glass tube (0.6 mm and the height of 2.8 mm) was filled with Cy5.5 solution (with the extinction coefficient of about 0.019 mm^−1^ *μM* ^−1^ and quantum efficiency of 0.23 at the peak excitation wavelength of 671 nm) [31]. The center of the target was determined at 21.1, 27.8, and 7.4 mm by the Micro-CT. The fluorescent yield of Cy5.5 was 0.0402 mm^−1^ [32]. For reconstruction, the CT data was segmented into five major anatomical components, including muscle, heart, lungs, liver, and kidneys.  Table 5 shows the optical properties of different organs [33].

**Figure 3:**
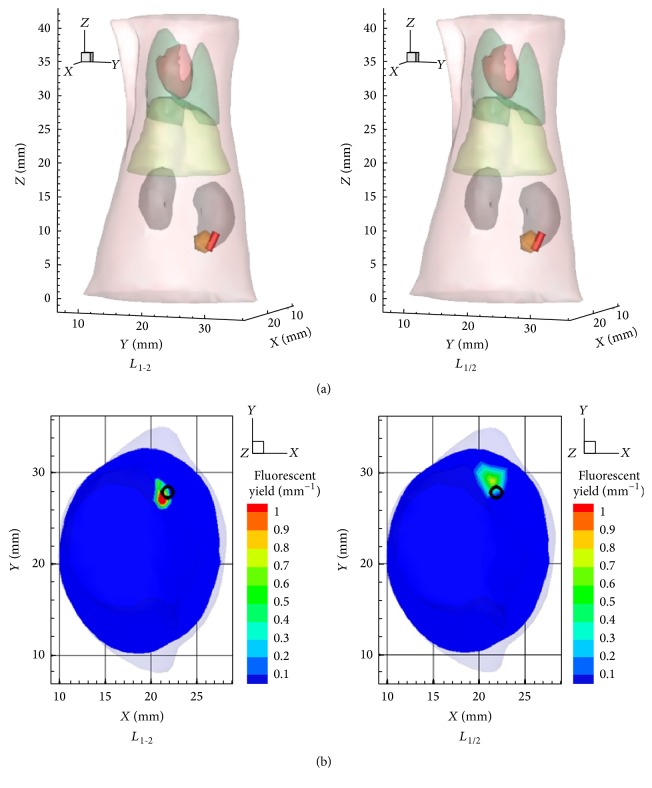
Reconstructed results* in vivo* experiment on adult BALB/C mouse. (a) The 3D view of the reconstructed results in which the red glass tube is the implanted fluorescent target and the green target denotes reconstructed results. (b) The 2D views (*Z* = 7.4 mm) of the reconstructed results. The black circle denotes the real positions of fluorescent target.

**Figure 4:**
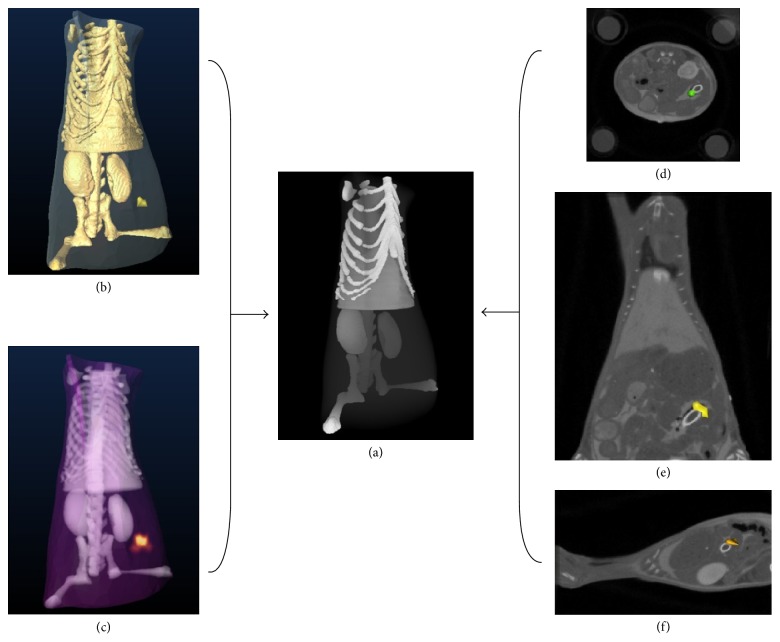
The reconstructed results of* in vivo* experiments via DCA-*L*_1-2_. (a) 3D rendering of the mouse. (b) The reconstructed result in 3D view. (c) The photon density distribution of the reconstructed result in 3D view. (d–f) The 2D views of the overlapped result with corresponding CT slices.

For inverse reconstruction, the segmented mouse torso data was discretized into a mesh with 3049 nodes and 14932 tetrahedral elements. Mutual coherence of the system matrix for inverse reconstruction was 99.87%. Comparison results between DCA-*L*_1-2_ and IRLS-*L*_1/2_ are shown in Table 6 and Figure 3. The 3D views of reconstructed results for* in vivo* experiments via DCA-*L*_1-2_ are shown in Figure 4.

## 4. Discussion and Conclusion

In this paper, novel *L*_1-2_ norm regularization was proposed to solve the inverse problem of FMT with highly coherent system matrix. To accurately recover the small fluorescent target, an iterative method based on DCA algorithm was presented to solve the nonconvex *L*_1-2_ minimization problem. And the ADMM method with an adaptive penalty was used to get fast convergence for the subproblem.

Simulated data on a 3D heterogeneous mouse model and* in vivo* experimental data acquired by a hybrid FMT/Micro-CT system were used to demonstrate the feasibility of the DCA-*L*_1-2_ algorithm for FMT. The comparative results of single target show that the DCA-*L*_1-2_ algorithm has better performance compared to other typical algorithms based on *L*_1/2_, *L*_1_, *L*_2_, and *L*_0_ norm regularizer. The robustness tests further illustrate that the DCA-*L*_1-2_ algorithm is stable and robust to measurement noise. In addition, decreasing the number of excitation sources from 18 to 4, DCA-*L*_1-2_ still yields satisfactory results.

However, the reconstructed fluorescent yield of the proposed method was still smaller than the true value. So new strategies that may further improve fluorescent yield will be our future research focuses. Moreover, we will also focus on investigating the multitargets resolution and new application of *L*_1-2_ norm regularizer in other imaging modalities in the near future.

In conclusion, both numerical experiments and* in vivo* experiments validated the good performance of *L*_1-2_ regularizer for FMT. Moreover, comparative experiments indicate that *L*_1-2_ outperforms the iterative reweighted strategies for *l*_*p*_ with *p* = 1/2 when system matrix is highly coherent.

## Figures and Tables

**Algorithm 1:**
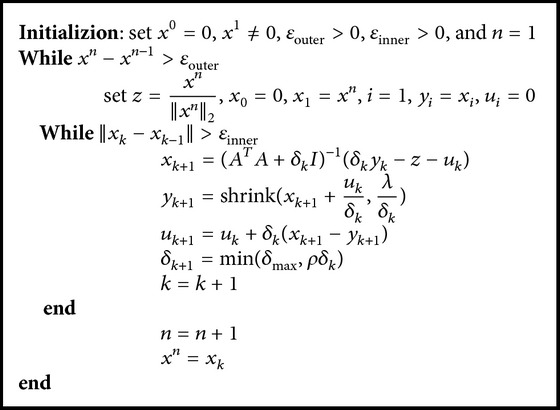
DCA-*L*_1-2_ algorithm for FMT.

**Table 1:** Optical parameters for the heterogeneous model.

Organs	*μ*_*a*,ex_ (mm^−1^)	*μ*_*s*,ex_′ (mm^−1^)	*μ*_*a*,em_ (mm^−1^)	*μ*_*s*,em_′ (mm^−1^)
Muscle	0.0052	1.08	0.0068	1.03
Heart	0.0083	1.01	0.0104	0.99
Lungs	0.0133	1.97	0.0203	1.95
Liver	0.0329	0.70	0.0176	0.65
Kidneys	0.0660	2.25	0.0380	2.02
Stomach	0.0114	1.74	0.0070	1.36

**Table 2:** Quantitative results in single target reconstruction experiment.

Methods	LE (mm)	Recon. FY (mm^−1^)	NRMSE (%)	PNZ (%)	Time (s)
DCA-*L*_1-2_	0.436	0.039	19	1.15	1.32
IRLS-*L*_1/2_	0.668	0.032	29	1.86	1.67
IVTCG-*L*_1_	1.279	0.005	67	2.47	31.74
Tikhonov-*L*_2_	2.169	0.004	79	11.96	21.96
OMP	1.135	0.004	63	9.83	1.21

**Table 3:** The results of DCA-*L*_1-2_ with different excitation sources.

The number of excitation sources	LE (mm)	Recon. FY (mm^−1^)	NRMSE (%)	PNZ (%)	Time (s)
18	0.436	0.039	19	1.15	1.67
12	0.497	0.034	21	1.28	1.32
8	0.518	0.029	23	2.34	1.23
4	0.614	0.014	36	3.73	0.89

**Table 4:** Impact of Gaussian noise on DCA-*L*_1-2_.

Noise level (%)	LE (mm)	Recon. FY (mm^−1^)	NRMSE (%)	PNZ (%)	Time (s)
5	0.436	0.039	19	1.15	1.67
15	0.437	0.039	19	1.15	1.67
25	0.437	0.038	19	1.15	1.72
35	0.514	0.038	20	1.33	1.78

**Table 5:** Optical parameters of the mouse model at 670 nm and 710 nm.

Organs	670 nm		710 nm	
	*μ*_*a*,ex_ (mm^−1^)	*μ*_*s*,ex_′ (mm^−1^)	*μ*_*a*,em_ (mm^−1^)	*μ*_*s*,em_′ (mm^−1^)
Muscle	0.075	0.412	0.043	0.350
Heart	0.051	0.944	0.030	0.870
Lungs	0.170	2.157	0.097	2.093
Liver	0.304	0.668	0.176	0.629
Kidneys	0.058	2.204	0.034	2.021

**Table 6:** Quantitative results of *in vivo* experiments.

Method	LE (mm)	Recon. FY (mm^−1^)	NRMSE (%)	PNZ (%)	Time (s)
DCA-*L*_1-2_	1.426	0.034	27	0.13	0.53
IRLS-*L*_1/2_	1.705	0.019	45	0.26	1.90
